# The BMP Antagonist Follistatin-Like 1 Is Required for Skeletal and Lung Organogenesis

**DOI:** 10.1371/journal.pone.0022616

**Published:** 2011-08-03

**Authors:** Marc Sylva, Vivian S. W. Li, Anita A. A. Buffing, Johan H. van Es, Maaike van den Born, Saskia van der Velden, Quinn Gunst, Jan Harm Koolstra, Antoon F. M. Moorman, Hans Clevers, Maurice J. B. van den Hoff

**Affiliations:** 1 Heart Failure Research Center, Academic Medical Center, Amsterdam, The Netherlands; 2 Hubrecht Institute, KNAW and University Medical Center, Utrecht, The Netherlands; 3 Academic Centre of Dentistry Amsterdam (ACTA), Amsterdam, The Netherlands; Ohio State University, United States of America

## Abstract

Follistatin-like 1 (Fstl1) is a secreted protein of the BMP inhibitor class. During development, expression of Fstl1 is already found in cleavage stage embryos and becomes gradually restricted to mesenchymal elements of most organs during subsequent development. Knock down experiments in chicken and zebrafish demonstrated a role as a BMP antagonist in early development. To investigate the role of Fstl1 during mouse development, a conditional Fstl1 KO allele as well as a Fstl1-GFP reporter mouse were created. KO mice die at birth from respiratory distress and show multiple defects in lung development. Also, skeletal development is affected. Endochondral bone development, limb patterning as well as patterning of the axial skeleton are perturbed in the absence of Fstl1. Taken together, these observations show that Fstl1 is a crucial regulator in BMP signalling during mouse development.

## Introduction

Bone morphogenetic protein (BMP) signalling is crucial for virtually all developmental processes [1]. BMPs were originally identified as inducers of ectopic bone formation *in vivo* [2]. Disruption of components required for canonical BMP signaling has demonstrated a role in skeletal development: deletion of either BMP ligands, their receptors or their downstream signaling molecules SMAD 1/5/8 results in diminished or absent endochondral bone formation [3]–[5]. Also, BMP is involved in limb bud patterning as a negative regulator of FGF expression in the apical ectodermal ridge [6].

For normal development, careful control of BMP signaling activity is required, with secreted BMP antagonists being essential regulators [7], [8]. Mice deficient for the BMP inhibitor Noggin show excessive cartilage formation and absence of joint formation [9]. Loss of Noggin can partially be rescued by haplo-insufficiency of Bmp4 [10]. Chordin-deficient mice display malformations of the axial skeleton as well as defects of the tracheal cartilage [11]. Gremlin is involved in limb bud patterning, its KO results in less digits and fused forearm bones [12]. Loss of Follistatin results in a decrease in the number of lumbar vertebrae and hypoplasia of the 13^th^ pair of ribs [13]. Taken together these findings underscore the importance of extracellular inhibitors of BMP signaling in normal development.

Follistatin-like 1 (Fstl1) is a BMP inhibitor. Its role in mouse development is unknown. Since its first identification [14], Fstl1 homologues have been isolated and found to be conserved down to ticks [15]. *In silico* analysis of Fstl1 identifies a domain similar to follistatin suggesting a role in TGFbeta super-family inhibition. The interaction of Fstl1 with TGFbeta super-family members is confirmed in Biacore analyses [16].

During development Fstl1 is already expressed in cleavage stage embryos and becomes gradually restricted to the mesenchyme of most organs [17]–[19]. Knock down of the chicken Fstl1 homologue, FLIK, results in reduction of paraxial mesoderm, perturbed dermamyotome specification and failure of neural induction, implying perturbation of Bmp signalling [20]. In zebrafish, Fstl1 is duplicated (fstl1a and fstl1b), loss of fstl1b in chordin-deficient embryos aggravates the ventralisation phenotype. This effect is comparable to loss of noggin in those embryos [19]. Knock down of both fstl1a and fstl1b results in an increase in chorda mesoderm [21]. This phenotype can largely be rescued by inhibiting bmp4 expression, suggesting an interaction between bmp4 and fstl1a/1b. This is further substantiated by the observation that BMP specific phosphorylated smad1/5/8 are decreased in fstl1a/1b deficient embryos Moreover, in vitro assays suggest that Fstl1 is able to inhibit Bmp4-mediated Smad-signalling [22]. Taken together *in vitro* and *in vivo* studies point to Fstl1 as an important BMP inhibitor during development.

To investigate the functional role of Fstl1 during development, we created a KO allele of Fstl1 as well as a GFP mouse line. Homozygous mice of both strains die at birth due to developmental malformations. Extensive skeletal and respiratory defect was observed in the Fstl1 mutant embryos similar to many other Bmp antagonists knockout phenotypes. Here we report that the Bmp antagonist Fstl1 is essential for embryonic skeletal and lung organogenesis. There is a recent publication during the preparation of this article where Geng and colleagues also demonstrated that Fstl1 affects lung development through suppressing Bmp4 signaling pathway [22]. Their data partially overlap with ours which lends further support to the important role of the Bmp antagonist Fstl1 in embryogenesis.

## Materials and Methods

All experimental procedures complied with national and institutional guidelines. The Institutional Welfare Committee of the University of Amsterdam and Utrecht University approved the generation, breeding, and analysis of the Fstl1^−/−^ and Fstl1^G/G^ lines, respectively. The approvals are registered as “DAE10484: Analyse van de rol van Follistatin-like 1 (Fstl1) tijdens de ontwikkeling van het embryo en het hart” for the Fstl1^−/−^ line and “HL10.1017: The role of Fstl1 in development and tissue homeostasis” for the Fstl1^G/G^ line.

**Figure 1:**
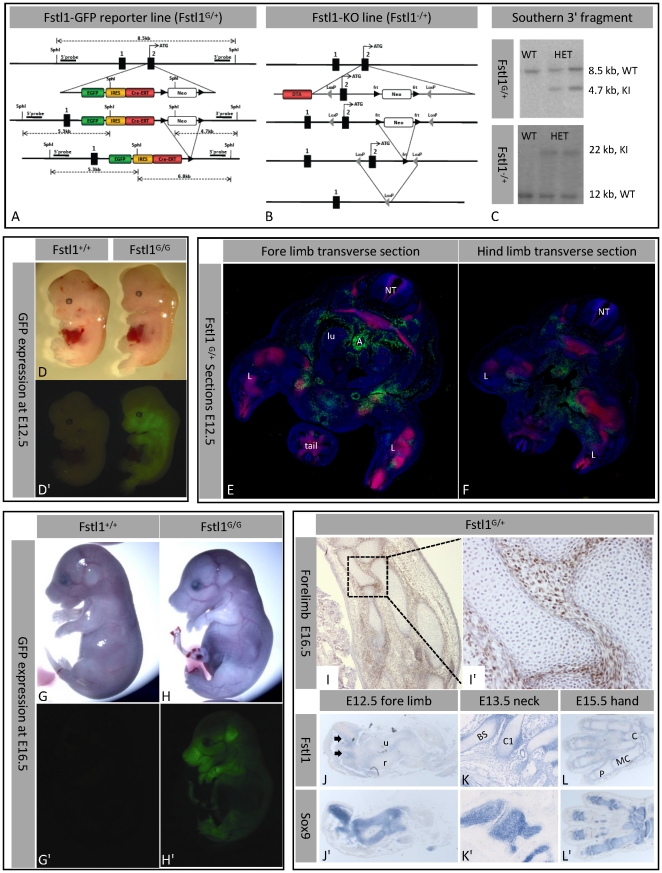
Generation of the transgenic mice and Fstl1 expression pattern. (A–C) Strategies for the generation of the transgenic mice. GFP expression (D′,G′,H′) in Fstl1+/+ and Fstl1G/G at E12.5 (D,D′) and E16.5 (G–H′). (E,F) Immunofluorescent staining showing GFP (green), Sox9 (red), and Dapi (blue) on sections of Fstl1G/+ embryos (NT = neural tube; l = limb; lu = lung; A = Aorta). (I) Immunohistochemistry showing GFP surrounding the long bones of the fore limb. Expression pattern of Fstl1 (J–L) and Sox9 (J′–L′) mRNA in adjacent sections. (arrow = interdigital space, u = ulna, r = radius, BS = Base of skull, C1 = Atlas, P = phalanxes, MC = metacarpals, C = carpals).

To generate the Fstl1^−/−^ (Fig. 1A,C), the 12965 bp Asp718I fragment containing Fstl1 sequences ranging from 6 kb upstream of exon 1 to 6.5 kb downstream of exon 2, was isolated from bacterial artificial chromosome RP23-1F14 (http://bacpac.chori.org). The 435 bp SacII-ApaLI fragment was subcloned and in the ApaI site located in intron1 the loxP site was inserted and sequence verified. The Asp718I-SacII and SacII-ApaLI fragments were inserted into pKOII [23] creating the 5′ and the 3′ flank by inserting the ApaL-Asp718I fragment. Vector sequences were removed and electroporated into V6.5 (C57Bl/6×129/Sv) stem cells. Clones were selected using diphtheria toxin and neomycin, and checked by PCR, Southern blotting, and karyotyping. Male chimeras were crossed with FVB females. Offspring was crossed with the FlpE mouse line [24] to remove the Neo-cassette and subsequently with the CMV-Cre line [25] to remove exon 2. This line is maintained on a FVB background. The Fstl1^−/−^ line was created and is breed in the animal facility of the University of Amsterdam.

To generate the Fstl1^G/G^, the EGFP-IRES-creERT2 cassette was inserted into the ATG of Fstl1 as previously described [26] (Fig. 1B,C). Fstl1 flanking arms were generated from 129S7-derived genomic BAC clones. The construct was electroporated into male 129/Ola-derived IB10 embryonic stem cells (provided by The Netherlands Cancer Institute). Clones were selected using neomycin, and checked by PCR, Southern blotting, and karyotyping. Male chimeras were crossed with C57BL/6 females. Offspring was crossed with PGK-Cre mice [27] to remove the neomycin-cassette. This line is maintained on a C57BL/6 background. The Fstl1^G/G^ line was created and is breed in the animal facility of the Hubrecht Institute.

### In situ hybridization

In situ hybridization was performed essentially as previously described [28]. Sectioned were deparaffinized, rehydrated in a graded series of alcohol and incubated with 10 mg/ml proteinase K dissolved in PBS for 15 min at 37°C. The proteinase K activity was blocked by rinsing the sections in 0.2% glycine in PBST (PBS+0.05%Tween-20) for 5 min. After rinsing in PBS, the sections were postfixed for 10 min in 4% PFA and 0.2% glutaraldehyde in PBS, followed by rinsing in PBS. After prehybridization for at least 1 hr at 70°C in hybridization mix (50%formamide, 5xSSC (20xSSC; 3 M NaCl, 0.3 M tri-sodium citrate, pH 4.5), 1% blocking solution (Roche), 5 mM EDTA, 0.1% 3-[(3-Cholamidopropyl) dimethylammonio]-1-propanesulfonate (Sigma; Steinheim, Germany), 0.5 mg/ml heparin (BD Biosciences; Erembodegem, Belgium), and 1 mg/ml yeast totalRNA (Roche), a digoxigenin (DIG)-labeled probe was added to the hybridization mix in a final concentration of 1 ng/ml. Probes specific to cardiac Troponin I (cTnI), Raldh1, Raldh2, Wt1, Tbx18, Snai1, Periostin and Fstl1 were used. After overnight hybridization, the sections were rinsed with 2xSSC, followed by two washes with 50% formamide, 2xSSC, pH 4.5, at 65°C, and rinsing in TNT (0.1 M Tris-HCl, pH = 7.5, 0.15 M NaCl, 0.05% Tween-20) at room temperature. Subsequently, the sections were incubated for 1 hr in MABT-block (100 mM Maleic Acid, 150 mM NaCl, pH 7.4, 0.05% Tween-20, 2% blocking solution), followed by 2 hours incubation in MABT-block containing 100 mU/ml alkaline phosphatase-conjugated anti-DIG Fab fragments (Roche catnr: 1093274). After rinsing in TNT and subsequently in NTM (100 mM Tris pH 9.0 100 mM NaCl, 50 mM MgCl_2_), probe binding was visualized using nitro blue tetrazolium chloride and 5-bromo-4-chloro-3-indolyl-phosphate (Roche catnr: 1681451). Color development was stopped by rinsing in double-distilled water. The sections were dehydrated in a graded ethanol series, rinsed in xylene, and embedded in Entellan. Images were recorded using a Leica DFC320 camera mounted on an AxioPhot microscope (Zeiss).

The coding sequence of mouse Fstl1 was PCR amplified, cloned in pBluescript SK+ (Stratagene), and sequence verified.

### Immunohistochemistry

Immunofluorescent staining was essentially performed as described [29]. In short, after deparaffinization and rehydration in a graded series of alcohol, the sections were boiled for 5 minutes in antigen unmasking solution (H3300, Vector), 15 min incubated in PBS+1% Triton-X100, and the signal was amplified with tyramide signal amplification (TSA NEL702, Perkin Elmer). The following primary antibodies were used: anti-Sox9 (Millipore, ab5535, 1∶1000), anti-SPC (Millipore, AB3786, 1∶250) and anti-GFP (Abcam, ab5450, 1∶200). For immunofluorescent visualization Alexa488 or Alexa568 conjugated goat-anti-rabbit and goat-anti-mouse antibodies (Molecular Probes; 1∶250) were used as secondary antibodies. Nuclei were visualized using Topro3 (Molecular Probes; 1∶500). Fluorescence was visualized using a Leica SPE confocal laser scanning microscope. For DAB staining a Horse-radish peroxidase conjugated anti-rabbit antibody (Envision) was used.

### Skeletal staining

Cartilage and bone were stained in embryos of various stages as previously described [30]. Embryos were fixed in 96% ethanol at room temperature overnight. Embryos of E17.5 and older were skinned before fixation. To stain the cartilage embryos were incubated in 80% ethanol, 20% glacial acetic acid and 150 mg/ml Alcian Blue 8GX (Michrome Edward Gurr Limited, London, UK) overnight. The embryos were incubated in 96% ethanol, that was replaced every 2 hours and the final incubation was overnight. Embryos of E14.5 and younger were passed to methanol in glass containers and the tissue was made translucent by incubation in a solution of Benzylbenzoate and Benzylalcohol (2∶1). Embryos of E15.5 and older were incubated with 2% KOH solution for 2 hours prior to overnight staining of the bones in 0.5% KOH and 50 mg/ml Alizarin Red S (BDH Chemicals). The staining solution was replaced by 0.25% KOH and replaced daily until the tissue was translucent. Subsequently, the embryos were passed to 30% glycerol for long term storage.

To visualize the calcified bones in neonatal mice microCT scans (µCT 40, Scanco Medical AG, Brüttisellen Switzerland) were made using standard settings. The obtained images were reconstructed in 3D using Amira and visualized using 3D-PDF [31].

## Results

### Neonatal lethality of Fstl1 deficient mice

**Figure 2:**
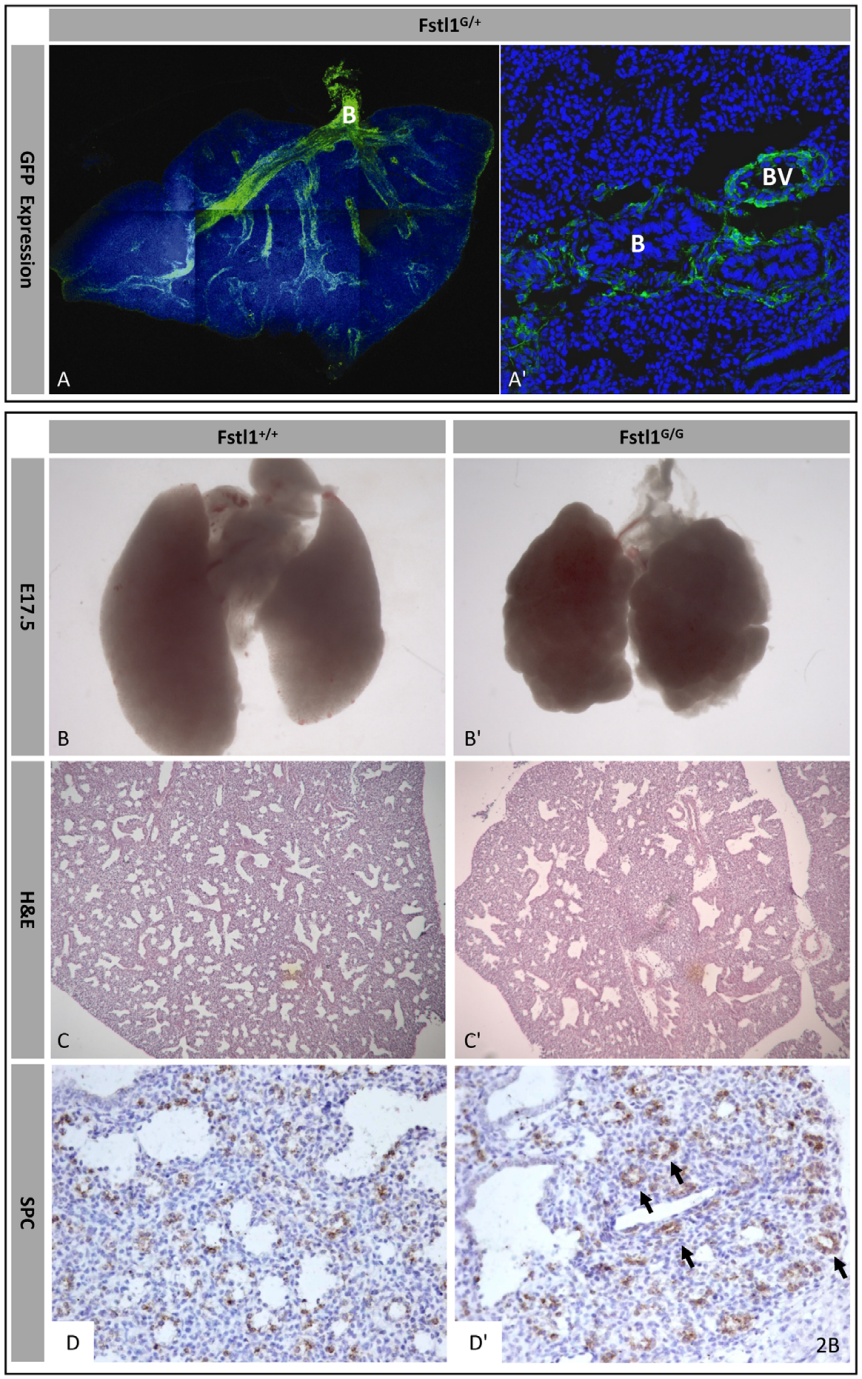
Lung phenotype. (A) Immunofluorescent staining of GFP (green) in E17.5 Fstl1G/+ lung. ((A) overview, (A′) detail, B = Bronchioles, BV = blood vessels). E17.5 lungs of Fstl1+/+ (B–D) and Fstl1G/G (B′–D′): (B,B′) gross morphology (C,C′) histology by H&E staining, and (D,D′) immunohistochemistry of surfactant-associated protein C (SPC).

Fstl1 knock out (KO) and GFP reporter alleles, Fstl1^−/+^ and Fstl1^G/+^ respectively, were generated. Both alleles predictably do not produce intact protein (Fig. 1A,B). The Fstl1^G/+^ mice allow us to visualize Fstl1 expression during embryogenesis *in vivo*, Fstl1 is strongly expressed in endothelium and mesenchyme of multiple organs throughout embryogenesis (Fig. 1. D–L). In developing limbs and axial skeleton Fstl1 mRNA is expressed complementary to the bone precursor marker Sox9 (Fig. 1I–L). Also in Fstl1^G/+^ mice, GFP-expressing cells do not overlap with Sox9-positive chondrocytes (Fig. 1E,F). This pattern does not change during development (Fig. 1J), suggesting a role in limb patterning. In lung, GFP-positive Fstl1 expressing cells are detected in mesenchyme surrounding airways as well as in endothelium of blood vessels (Fig. 2A).

Both Fstl1^−/+^ and Fstl1^G/+^ heterozygous strains show no obvious defects. In both strains homozygous mice are found in normal Mendelian ratios up to embryonic day (E) 18.5. However, at neonatal day 0 no living KO mice are retrieved. At birth Fstl1 KO mice change colour from pink to purple while gasping and die within minutes, suggesting a respiratory defect.

### Tracheomalacia and lung differentiation defects in Fstl1 mutants

To investigate the cause of neonatal lethality of Fstl1 KO mice, the respiratory tract was analyzed. No inflation of the lung is detected in any of the Fstl1^G/G^ dead mice (data not shown), documenting the inability to breath of Fstl1 KO mice upon birth.

**Figure 3:**
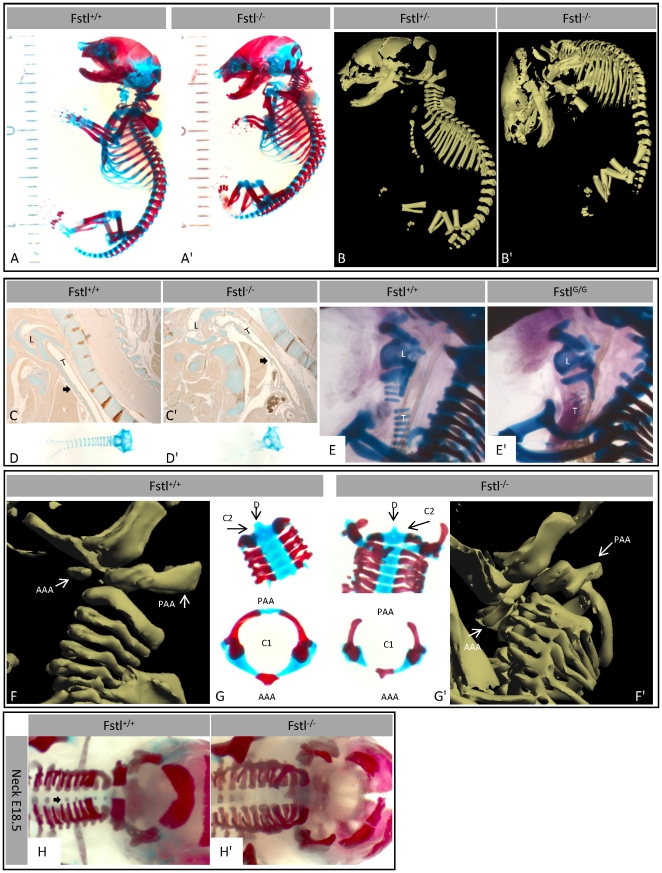
Skeletal phenotype I. Skeletal staining (A,A′) and microCT scans (B,B′) of E18.5 embryos. Skeletal staining of sagital sections (C,C′), isolated trachea (D,D′) and close-up (E.E′) of the trachea in situ (L = Larynx, T = Trachea, arrows =  tracheal cartilage). (F,F′) Lateral views of microCT scans of the neck. (G,G′) Skeletal staining of the cervical vertebrae (C1 = Atlas, AAA = Anterior Arch of the Atlas, PAA = Posterior Arch of the Atlas, C2 = Axis, D = dens axis). (H,H′) Alizarin red staining of the neck (arrows = ossification centers).

Skeletal staining on whole-mounts and tissue sections reveals both ill spaced and hypoplastic tracheal rings (Fig. 3C–E), explaining the respiratory distress and subsequent death. Comparison of the late embryonic lung morphology shows irregular bubble-shaped lobes in Fstl1^G/G^ lungs instead of the typical pyramidal shape of the wild-type and heterozygous lobes (Fig. 2B). Histological analyses of E17.5 lungs reveal abnormal patterning of proximal and distal airway epithelium. Fstl1 deficient lungs display irregular, enlarged proximal bronchioles and smaller, enclosed distal sacs (Fig. 2C). Immunostaining for the distal respiratory tract specific epithelial marker pulmonary surfactant-associated protein C demonstrates a tight, focal structure of the distal alveolar airspaces in Fstl1^G/G^ lungs compared to wild-type (Fig. 2D).

### Axial skeletal defects

At E18.5 KO embryos are smaller than their wild type littermates (Fig. 3A,B). Skeletal preparations showed abnormal curvature of the spine, with increased cervical lordosis and lumbar kyphosis. Additionally, the head is displaced ventro-caudally and positioned in front of the neck. To study the neck in detail micro-CT scans were performed and reconstructed in 3D (Fig. 3B, S1, S2), showing ventro-caudal displacement of the atlas; its anterior arch is positioned in front of the cervical spine (Fig. 3F,G). The posterior part of the cervical vertebrae is missing along with the attachment of the anterior arch of the atlas to its posterior arch. Notably the dens axis is present in the KO situation. Moreover, reduced ossification of the vertebral bodies of the spine is observed in KO mice (Fig. 3H).

### Limb defects

**Figure 4:**
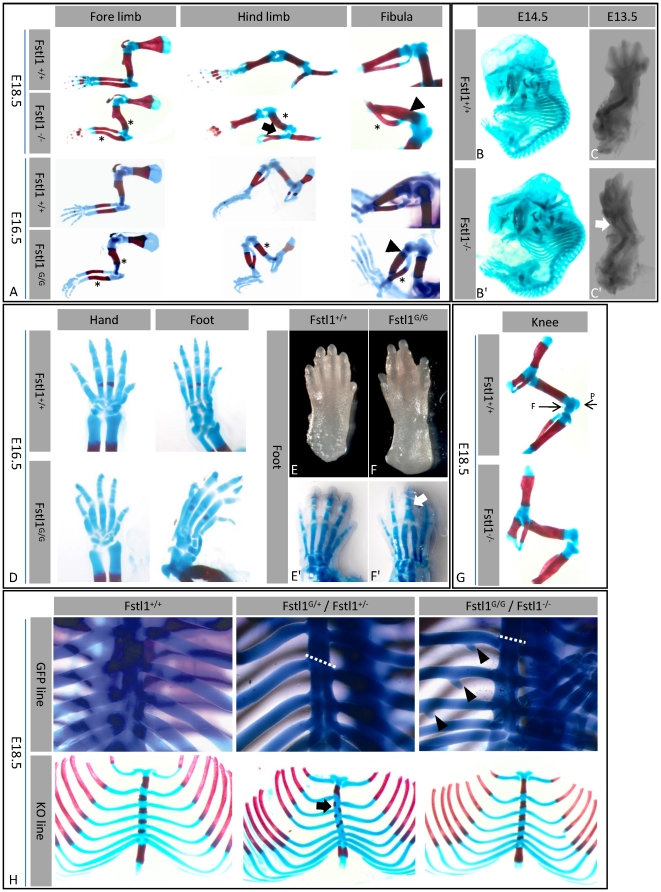
Skeletal phenotype II. (A) Limbs of E18.5 and E16.5 embryos (asterisk = bend bones, arrow = displaced hip, arrow head = abnormal fibula attachment). (B,B′) Skeletal staining of E14.5 embryos showing abnormal spine curvature and bend radius (arrow) in E13.5 (C,C′). (D) Delayed ossification and curved digits in Fstl1^G/G^ mice. (E–F′) Digital fusion (arrow) in Fstl1^G/G^ mice. (G) Absence of patella (P) and fabella (F) in Fstl1^−/−^. (H) Rib cages of wild type, heterozygous, and homozygous mice, showing asymmetrical rib-sternum attachments (dotted line and arrows) and rib processes (arrow head).

In skeletal preparations, bending of the radius and ulna (known as campomelia) as well as of the humerus, femur and fibula are observed in Fstl1 KO mice (Fig. 4A). Instead of running in parallel to the tibia, the fibula is curved and its proximal attachment is displaced from lateral to medial (Fig. 4A). Probably due to this abnormal alignment of the fibula, twisted hind limbs are frequently observed in KO mice. In addition some KO mice display hip displacement and all KOs show absence of the patella and fabella (Fig. 4G). In addition to the defects in the long bones, Fstl1^G/G^ mice show several digit abnormalities. These include the delayed ossification of the metacarpals, distorted and irregular alignment of the digits, as well as fusion of digits at the site of the proximal phalanxes (Fig. 4D–F).

### Skeletal defects arise early in development

To assess at what time point absence of Fstl1 results in aberrant skeletal development, skeletal preparations were prepared down to E13.5. At E14.5 the KO embryos already show abnormal spine curvature, most prominent in the cervical part (Fig. 4B). Moreover, campomelia is already present at E13.5 (Fig. 4C). These observations demonstrate a role for Fstl1 early in endochondral bone development prior to ossification. Comparison of the expression patterns of genes involved in early skeletal development, ie Prrx1, Sox9, Col2A1 and Col10a1, did not identify marked differences between KO and WT embryos at E12.5 and E15.5 (Fig. S3).

### Defects in rib sternal attachment

Close examination of the skeleton of heterozygous KO mice identified asymmetrical attachments of the ribs to the sternum and shifted ossification centers, known as rib-sternum mispairing (Fig. 4H). No asymmetry is observed at the site of rib-vertebra attachment, nor other evidence of homeotic transformations. In homozygous KO mice the rib-sternum mispairing is not as evident as in heterozyguous KO mice. In homozygous KO mice the ossification centers are not perpendicular to the sternum, but show an angle. An additional abnormality of the ribs is present in the homozygous Fstl1^G/G^ mice, displaying cartilaginous processes at the ventral part of the first five ribs (Fig. 4H).

## Discussion

The function of Fstl1, a glycosylated and secreted protein, is pooly understood. In vitro and in vivo studies suggest that Fstl1 belongs to the class of secreted BMP inhibitors. To evaluate the functional role of Fstl1, a conditional KO allele as well as a GFP knock in mouse were created. Though Fstl1 is expressed from as early as gastrulation in the developing embryo, homozygous KO embryos are found in expected mendelian ratio up to the end of gestation. However, at birth the homozygous KO embryos die in respiratory distress and are devoured by the mother. This is observed for both mouse strains.

### Respiratory phenotype

The observed respiratory distress and subsequent death is due tracheomalacia and immature distal alveolar development. This lung phenotype resembles the recently reported phenotype in another Fstl1 KO mouse [22]. In the latter study it is shown that the lung phenotype can be rescued in vitro by the addition of the BMP inhibitor Noggin. These findings suggest that loss of the BMP antagonist Fstl1 disrupts the delicate balance of BMP and FGF signalling that is essential for normal respiratory tract development, in particular proximal-distal lung epithelial patterning [32].

### Fstl1 functions in concert with other BMP inhibitors

The retrieval of Fstl1 KO embryos up to the end of gestation at Mendelian ratios is remarkable, because in chicken and zebra fish loss of Fstl1 results in perturbed mesoderm development. [19], [20]. Based on the observations in zebra fish and chicken it was expected that the Fstl1 embryos would die early during development as a result of disrupted mesoderm formation. The observed fenotype in in chicken and zebra fish is attributed to insufficient inhibition of BMP signalling. Taken together these finding suggest that in mice other BMP inhibitors are redundant, resulting in survival of Fstl1 KO embryos up to the end of gestation. In line with this idea, it was observed that in zebra fish disruption of Fstl1 can enhance defects in BMP inhibitor Chordin deficient embryos. Like Fstl1 KO mice, Chordin KO mice suffer from defects of the cervical vertebrae most markedly the atlas and abnormal development of the tracheal cartilage rings [11]. The overlap in phenotype between Chordin and the here reported Fstl1 deficient mice suggests that, like in zebrafish [19], Fstl1 functions in concert with Chordin.

### Digital defects

During limb bud development, Bmp signaling is important in regulating apical ectodermal ridge (AER) formation and interdigital apoptosis through an epithelial-mesenchymal feedback loop [33], [34]. Many Bmp antagonist-deficient mouse models display multiple distal limb defects, including loss or fusion of digit formation [12]. In man, a genetic disorder called multiple synostosis syndrome is characterized by multiple bone fusions. This syndrome is associated with a gain-of-function mutation of GDF5 (BMP14), rendering it resistant to inhibition by Noggin [35]. The digit defects observed in the Fstl1^G/G^ mice resemble the clinical limb phenotypes of multiple synostosis syndrome, and those observed in Bmp inhibitor deficient mice, suggesting that Fstl1 affects digital limb formation through the Bmp signaling pathway.

### Loss of Fstl1 ties Bmp signaling to campomelia

Besides the digit abnormalities both Fstl1 KO strains display bending of the humerus, ulna and radius in the fore limb and of the femur and fibula in the hind limb, as well as absence of the patella and fabella, and to a lesser extend hip displacement.

The bending of the bones in the fore limb is reffered to as campomalia. In man and mice campomelia is caused by heterozygous loss of the transcription factor Sox9. Interestingly, after heterozygous Sox9 deletion besides camptomelia also hip displacement, hypoplasia of the patella, and tracheomalacia was reported [36], [37]. Although perturbed BMP signaling in mice is associated with many different limb phenotypes [3], [9], [12], campomelia has not been reported. From conditional deletion analysis of Sox9, it is known that early deletion using Prrx1-Cre results in campomelia [38]. Moreover, deletion of the transcription factor Prrx1 itself, which is expressed prior to Sox9, results among other skeletal defects also in campomelia [39]. This implies that the patho-physiological mechanism for bending of the long bones takes place early in development. Interestingly, in Fstl1 KO mice, skeletal defects are observed as early as E13.5 demonstrating an early role for Fstl1 in limb development. Taking together, these findings point to a role for BMP signaling in the development of campomelia.

### Axial patterning defects

Upon Fstl1 disruption rib-sternum mispairing is observed which is most evident in the heterozygous Fstl1 KO as the ribs are most prominently malaligned. The mechanism underlying rib-sternum mispairing is poorly understood, but thought to arise either from asymmetrical fusion of the sternal bands or from asymmetrical migration of ribs towards these bands [40]. Rib-sternum mispairing is observed in different KO mouse strains [40]–[42], being part of homeotic transformations as observed in the Hoxa5 mutants [42], and/or as a result of disrupted BMP signaling. Interestingly, homeotic transformations are part of the phenotype when BMP signaling is disrupted. Gdf11(Bmp11) deficient mice show extensive homeotic transformations of the axial skeleton and a posterior shift of Hox gene expression [43]. Other homeotic transformations can be observed in Follistatin KO mice, including absence or hypoplasia of the 13^th^ rib as well as loss of the sixth lumbar vertebra [13]. In Bmp5-deficient mice the 13^th^ rib is absent [44]. In a heterozygous Noggin background, deficiency of the BMP inhibitor Dan results in a posterior transformation of the last lumbar vertebra [45]. Also in Bmp7 KO mice rib-sternum mispairing is observed [46]. Although it seems contradictory that deletion of Bmp7 gives rise to a phenotype similar to deletion of the Bmp inhibitor Fstl1, it does show that BMP-mediated signaling is involved in the process of rib-sternal attachment. Of note, some BMP inhibitors, like twisted-gastrulation, can function under certain conditions as stimulators of BMP signaling [47].

In addition to the rib-sternum mispairing cartilaginous processes were observed on the ventral side of the ribs in Fstl1^G/G^ mice. These processes were never reported before in mice, but uncinate processes of ribs are a normal part of the skeleton of crocodiles, birds and some dinosaurs. Interestingly, although the uncinate processes are situated at the dorsal side of the rib cage, these findings could be interpreted as an atavistic event.

### General conclusions

Deletion of Fstl1 results in extensive skeletal defects and neonatal lethality due to respiratory defects. These defects demonstrate a role for the BMP inhibitor Fstl1 in lung development, endochondral bone formation, limb patterning, and patterning of the ribcage. These observations add Fstl1 to the extensive pallet of BMP modulators regulating organogenesis. Typically, deletion of Bmp antagonists during mouse embryonic development are lethal and cause multiple skeletal and lung defects. Thus, Bmp signaling is under very precise homeostatic regulation during embryonic development. Taking the broad expression pattern of Fstl1 into account, Fstl1 might play a role in the development of other organs. For the analysis of those developmental processes as well as the disease models in which Fstl1 is implicated, the newly generated conditional KO mice as well as the GFP reporter mice should be valuable tools.

## Supporting Information

Figure S1 **3D reconstruction of a microCT scan of an E18.5 wild type embryo.**
(PDF)

Figure S2 **3D reconstruction of a microCT scan of an E18.5 Fstl1^−/−^embryo.**
(PDF)

Figure S3 **Skeletogenesis marker gene expression.** RNA in situ hybridization of E12.5 and E15.5 fore limbs showing similar expression patterns of Prrx1, Sox9, Col2A1, and Col10A1 mRNA in wild type (Fstl1^+/+^) and knockout (Fstl1^−/−^) embryos.
(TIF)
