# Supplementary figures and images for: The BMP Antagonist Follistatin-Like 1 Is Required for Skeletal and Lung Organogenesis
Source: PLoS One. 2011 Aug 3;6(8):e22616. doi: 10.1371/journal.pone.0022616 (PMC3149603; doi:10.1371/journal.pone.0022616)

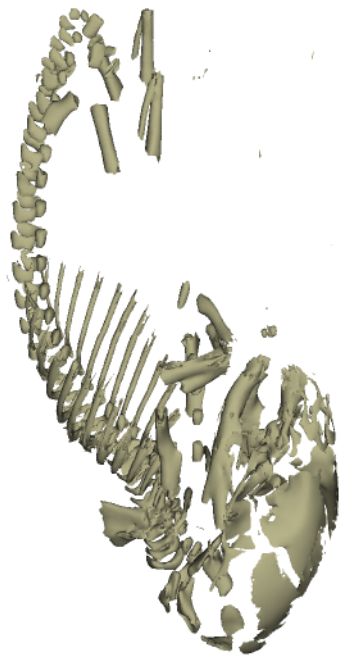

Supplement: Figure S1 — 3D reconstruction of a microCT scan of an E18.5 wild type embryo. (PDF) [file pone.0022616.s001.pdf]

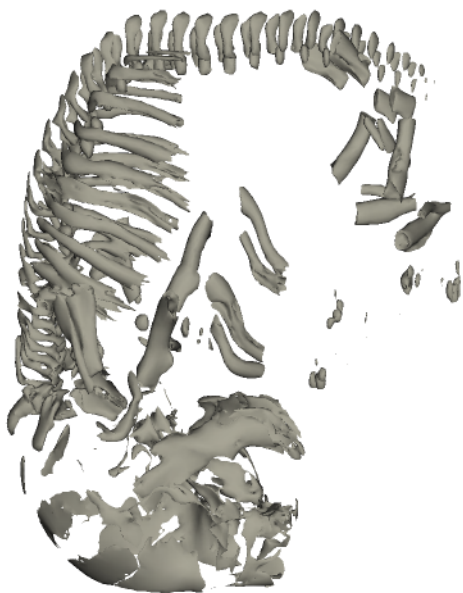

Supplement: Figure S2 — 3D reconstruction of a microCT scan of an E18.5 Fstl1−/−embryo. (PDF) [file pone.0022616.s002.pdf]

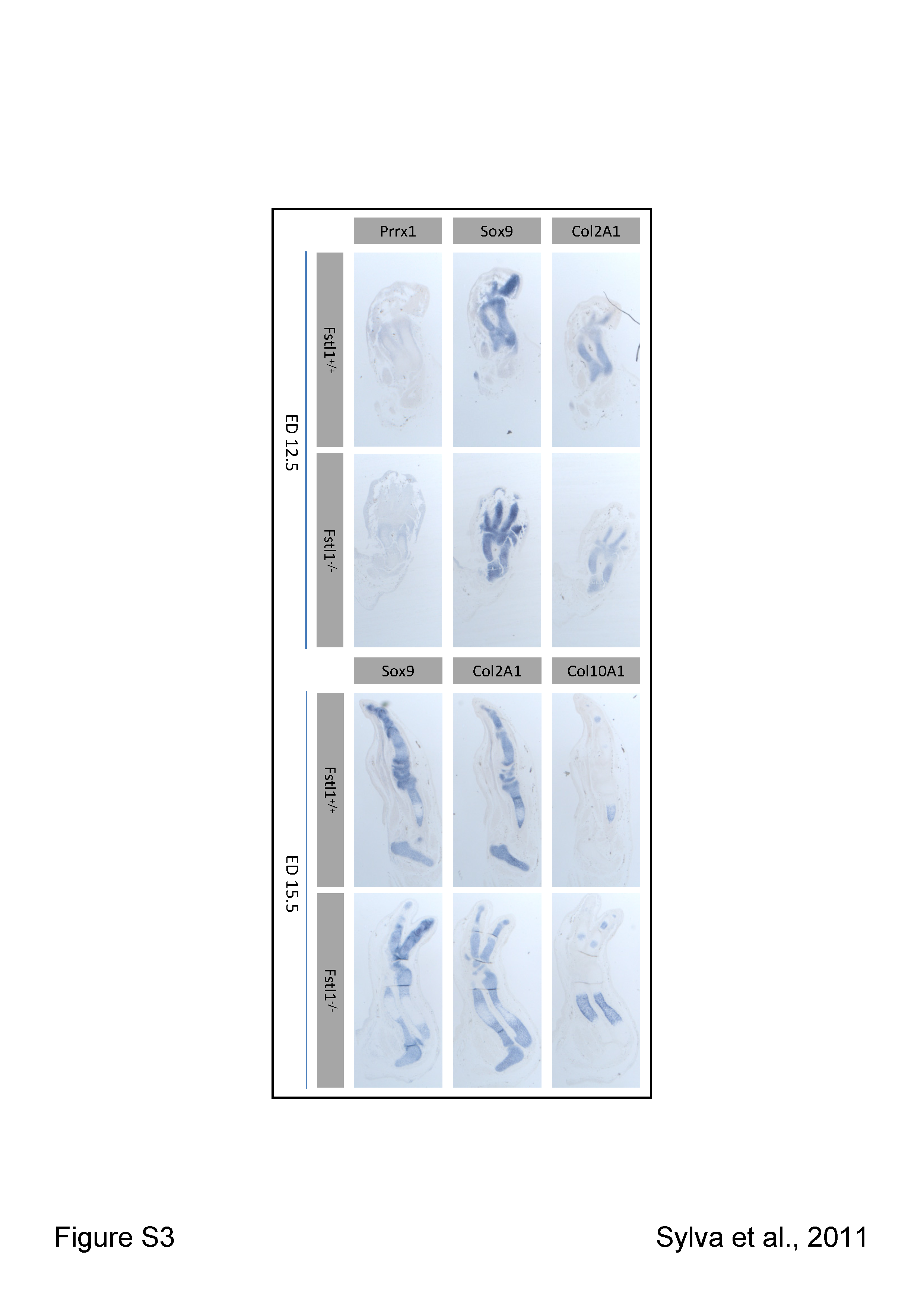

Supplement: Figure S3 — Skeletogenesis marker gene expression. RNA in situ hybridization of E12.5 and E15.5 fore limbs showing similar expression patterns of Prrx1, Sox9, Col2A1, and Col10A1 mRNA in wild type (Fstl1+/+) and knockout (Fstl1−/−) embryos. (TIF) [file pone.0022616.s003.tif]
